# Supplementary material for: Novel Long-Acting Oxytocin Analog with Increased Efficacy in Reducing Food Intake and Body Weight
Source: Int J Mol Sci. 2022 Sep 24;23(19):11249. doi: 10.3390/ijms231911249 (PMC9569447; doi:10.3390/ijms231911249)
Supplement: Supplementary file 1 [file ijms-23-11249-s001.zip › ijms-1905256-supplementary.pdf]

Supplemental data

Exploratory study using quantitative real-time PCR for studying gene expression of thermoregulatory proteins

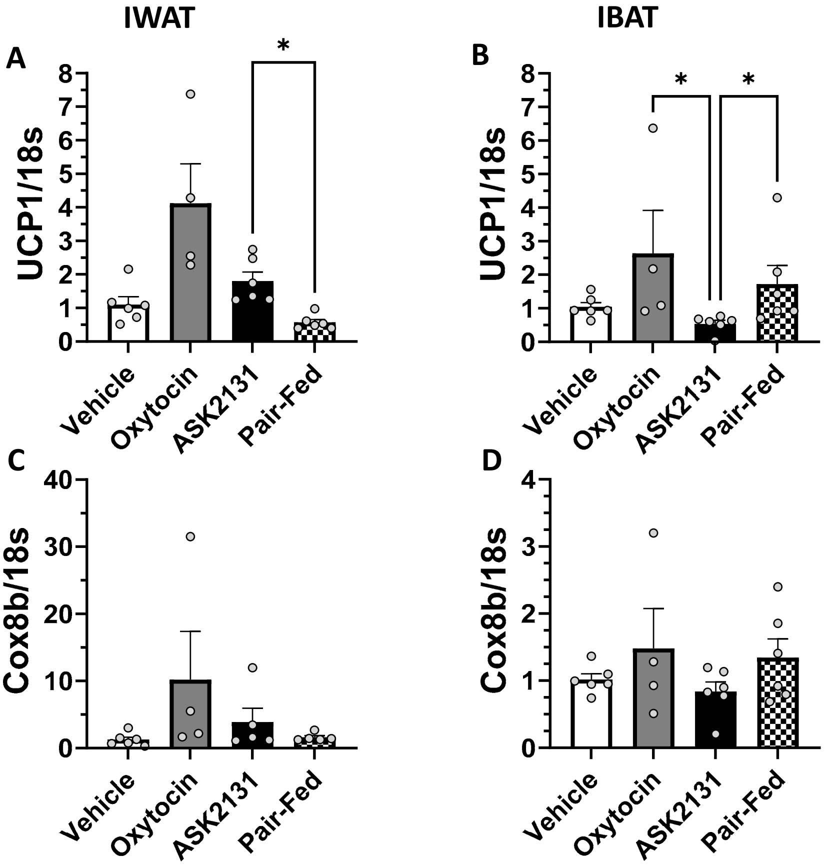

Supplementary Figure S1. Inguinal white adipose tissue (IWAT) and interscapular brown adipose tissue (IBAT) were collected from diet-induced obese rats treated with vehicle, oxytocin, ASK2131, and the pair-fed to the ASK2131-treated rats. Gene expression (mRNA levels) determined by qRT-PCR for UCP1 (**A**,**B**) and Cox8b (**C**,**D**). Individual data points are indicated (○); * $p < 0.05$
